# HNP-1 and HBD-1 as Biomarkers for the Immune Systems of Elite Basketball Athletes

**DOI:** 10.3390/antibiotics9060306

**Published:** 2020-06-07

**Authors:** Raffaela Pero, Mariarita Brancaccio, Cristina Mennitti, Luca Gentile, Adelaide Franco, Sonia Laneri, Margherita G. De Biasi, Chiara Pagliuca, Roberta Colicchio, Paola Salvatore, Giovanni D’Alicandro, Daniela Terracciano, Michele Cennamo, Evelina La Civita, Antonietta Liotti, Cristina Mazzaccara, Giulia Frisso, Barbara Lombardo, Olga Scudiero

**Affiliations:** 1 Department of Molecular Medicine and Medical Biotechnology, University of Naples Federico II, Via S. Pansini 5, 80131 Naples, Italy; cristinamennitti@libero.it (C.M.); chiara.pagliuca@unina.it (C.P.); roberta.colicchio@unina.it (R.C.); paola.salvatore@unina.it (P.S.); cristina.mazzaccara@unina.it (C.M.); giulia.frisso@unina.it (G.F.); barbara.lombardo@unina.it (B.L.); 2 Task Force on Microbiome Studies, University of Naples Federico II, 80100 Naples, Italy; 3 Department of Biology and Evolution of Marine Organisms, Stazione Zoologica Anton Dohrn, Villa Comunale, 80121 Naples, Italy; mariarita.brancaccio@szn.it; 4 Ceinge Biotecnologie Avanzate S. C. a R. L., 80131 Naples, Italy; gentilelu@ceinge.unina.it; 5 Department of Pharmacy, University of Naples Federico II, 80131 Naples, Italy; ade.franco@studenti.unina.it (A.F.); slaneri@unina.it (S.L.); margherita.debiasi@unina.it (M.G.D.B.); 6 Department of Neuroscience and Rehabilitation, Center of Sports Medicine and Disability, AORN, Santobono-Pausillipon, 80122 Naples, Italy; ninodalicandro@libero.it; 7 Department of Translational Medical Sciences, University of Naples “Federico II”, 80131 Naples, Italy; daniela.terracciano@unina.it (D.T.); michele.cennamo@unina.it (M.C.); evelina.lacivita@unina.it (E.L.C.); tonialiotti@virgilio.it (A.L.)

**Keywords:** human defensins, physical activity, immune system, stress hormones

## Abstract

Acute or strenuous exercise is sometimes related to upper respiratory tract infections in athletes. Practicing intense and regular exercise can lead to incorrect activation of the immune system, causing athletes to be excluded from training programs and competitions. Defensins are small antimicrobial peptides that are part of the innate immune system and dynamically involved in several biological activities. In this study, we highlight the role of human defensins in competitive basketball athletes. In particular, we consider the behavior of alpha- and beta-defensins together with white blood cells in a cohort of players. Moreover, we focus our attention on cortisol, a physiological indicator of stress, and testosterone, both of which are human hormones involved in muscle metabolism. The free-testosterone/cortisol ratio is considered to be an indicator of overtraining among athletes. This paper provides an up-to-date information of the role of human defensins as self-defense molecules during a continuous stressor such as long-term exercise, and it recognizes them as potential markers of infection.

## 1. Introduction

Several studies have suggested that physical activity induces considerable physiological changes within the immune system [1,2,3,4,5,6]. Human defensins are small peptides (29–34 amino acids) that belong to the human immune system. They represent a significant family of antimicrobial peptides (AMPs) that play a crucial role in infection and inflammation and that act against infectious pathogens (bacteria, viruses, fungi, and parasites) [7]. Human defensins are organized into two classes, alpha and beta [8], based on the distance between the cysteine residues and the topology of the disulfide bridges. Human alpha-defensins are small molecules isolated from human blood, and they are mainly expressed by neutrophils [9,10]; as a result, they are called human neutrophil peptides (HPN1-4). Alpha defensin 1 (HPN1) is a constitutively expressed defensin, although its levels increase during infection. Human alpha defensins 5 and 6 (HD5 and HD6) are isolated from human Paneth cells [11,12]; these molecules are only expressed in the Paneth cells of human intestines and the female reproductive system [13]. In contrast, human beta-defensins (HBDs) are mainly found in association with epithelial surfaces (skin, gut, trachea, and oral epithelia). In humans, the most studied beta-defensins are HBD1–4 [14]. Furthermore, it is known that beta defensin 1 (HBD1) is constitutively expressed; however, its serum levels may increase in the presence of diseases such as cystic fibrosis, in which it plays a crucial role in the innate immunity of the mucous membrane in the lung [15,16]. Moreover, both alpha- and beta-defensins have broad antibacterial activity [17,18] and multiple roles in innate immunity.

Physical exercise is known to cause an increase in IgA levels in athletes’ immune systems [19]. In addition, preservation of the mucosa in professional athletes reduces the initiation of upper respiratory tract infections (URTIs). URTIs can harm athletes and negatively affect competitions and training. In fact, recent studies have showed that intense exercise was associated with self-reported symptoms of URTIs in athletes [20,21,22].

In recent years, URTIs have aroused considerable interest in the medical and scientific fields due to the development of strong resistance to the antibiotics generally used to treat these disorders, including *Streptococcus pneumonia*, *Haemophilus influenza, Moraxella catarrhalis, Streptococcus pyogenes,* and *Staphylococcus aureus,* which are the pathogens most frequently found in URTIs. These five pathogens exhibit strong resistance to beta-lactam and macrolide antibiotics. However, the therapy currently in use involves intramuscular administration of beta-lactams and macrolides, such as amoxicillin/clavulanate or ceftriaxone. Alternative therapies involve the administration of quinolones, but these therapeutic agents are reserved for patients who develop resistance or allergies to beta-lactams and macrolides [23,24,25].

Several studies have highlighted a strong relationship between beta-defensins and the appearance of various respiratory disorders [26]. Changes in beta-defensin expression levels can lead to the improvement or deterioration of a disease. HBDs represent a valid and effective treatment against pathologies caused by drug-resistant microorganisms; in addition, HBD administration represents a solid alternative to the pharmacological therapies currently in use [27]. Moreover, a diet rich in immune nutrients (arginine, glutamine, dietary nucleotides, and fish oil) regulates the expression of HBD1 by improving immune-system function by supporting the reduction of infectious diseases and the use of antibiotics, as well as hospitalization [28]. Gałązka-Franta and colleagues reported that URTIs in competitive athletes require considerable attention, and that diagnostic methods and therapies should be used to maintain their levels of activity while following the antidoping rules currently in place [29].

A few studies have investigated the levels of alpha- and beta-defensins following physical exercise. Some studies reported an increase in the salivary levels of beta-defensins (HBD2) following exercise and an accompanying increase in hNP1–3 levels, thereby enhancing immune function [5,9]. Generally, physical exercise elicits the initial activation of leukocytes [24,25,26,27,28,29]. Specific temporal patterns exist for specific subpopulations of leukocytes involved in the post-exercise state, including a rise in neutrophils and lymphocyte migration after exercise. Hence, the neutrophil/lymphocyte ratio (NLR), which can be measured easily from whole blood, can be used as an indication of the magnitude of the systemic inflammation and the severity of the muscle damage incurred by a given bout of exercise [30]. Another important factor involved in an athlete’s physical status is cortisol. Cortisol regulates the exercise-induced acute inflammatory response and was previously reported to be associated with a rise in neutrophil numbers and a suppression of lymphocytes [31]. Furthermore, the magnitude and function of the circulating cells in the innate immune system, such as neutrophils and natural killer cells (NK-cells), and the acquired immune system may be altered by acute and chronic exposure to exercise [3]. Blood lymphocyte numbers were shown to exponentially increase following an acute bout of exercise [32,33]. Lymphocytes are stress-responsive cells that are mobilized during physical exercise and psychological stress; therefore, analysis of the proliferation of these cells in athletes suffering symptoms consistent with this phenomenon would be of interest [32]. Cortisol represents a physiological indicator of stress [34]. Sports activity can induce physical stress, causing increased secretion of cortisol from the adrenal cortex [35]. It has been suggested that cortisol levels indicate physical stress in competitive athletes and that increasing cortisol levels through exercise can trigger immunosuppression [36,37,38]. Previous studies have shed light on how increased glucocorticoid levels decrease HBD-2 expression [38,39,40], emphasizing that this relationship could be evaluated in biological fluids such as saliva [5]. The adaptation of the endocrine system to exercise training occurs physiologically via the buffering of anabolic and catabolic processes [2,41]. For example, altered cortisol/testosterone ratios, an indicator of anabolic–catabolic balance, were utilized with limited success to determine the physiological strain of exercise training [42]. The free-testosterone/cortisol ratio (fr-T/C) was proposed as a major indicator of anabolic and catabolic effects resulting from overtraining, especially among athletes. A reduction of the testosterone/cortisol ratio by more than 30% is related to overtraining status [43]. Moreover, Urhausen et al. reported that significant increases in testosterone levels without similar big differences in cortisol levels occurred after maximum aerobic training [44]; this variation in adrenal hormones may have been related to subjects responding differently to exercise [45]. While cortisol has a catabolic effect, testosterone is responsible for the stimulation of the anabolic process of skeletal muscle growth, which increases linearly in response to exercise [46,47,48,49,50,51].

Therefore, this study aimed to examine the responses of basketball players during training, competition, and recovery, considering (i) the immune system, i.e., (a) mucosal immunity (human defensins: HNP1 and HBD-1) and (b) differences in exercise-induced leucocyte subset numbers and total leucocytes, and (ii) stress hormones, such as cortisol and testosterone.

We hypothesized that physical activity would increase serum alpha- and beta-defensins, thereby enhancing immune function. This might be useful for the maintenance of older adults’ and athletes’ health.

## 2. Results

### 2.1. Evaluation of α- and β-Defensins in Professional Athletes

**Figure 1:**
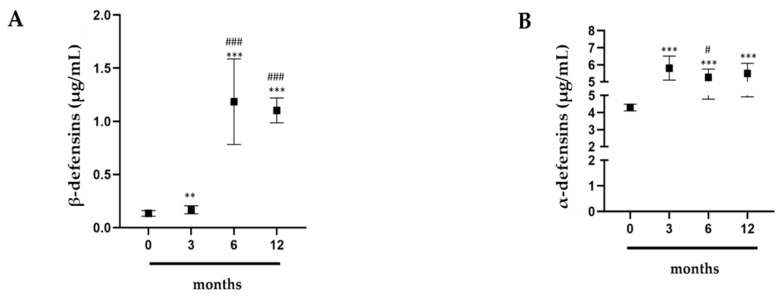
ELISA assay. (**A**) Beta-defensin and (**B**) alpha-defensin levels of 15 professional basketball athletes at 0, 3, 6, and 12 months since the start of a championship competition. The data are expressed as the means ± SDs. Significance was determined by the Student’s *t*-test: (*p* < 0.05), ** (*p* < 0.01), and *** (*p* < 0.001) represent significance compared to 0 months; ^#^ (*p* < 0.05), (*p* < 0.01), and ^###^ (*p* < 0.001) represent significance compared to 3 months.

To determine how intense physical activity influences antimicrobial peptide (AMP) trends, alpha- and beta-defensins (HNP1 and HBD-1, respectively) were evaluated with ELISA (Enzyme-Linked ImmunoSorbent Assay) using the athletes’ sera. The ELISA test showed that levels of beta-defensins were higher between 3 and 12 months compared to 0 months (Figure 1A). We also compared 3 months against 6 and 12 months, which showed a significant increase (Figure 1A), and 6 months against 12 months (Figure 1A). The levels between 6 and 12 months were constant (Figure 1A). The alpha-defensins exhibited a similar trend (Figure 1B). In fact, their levels were higher between 3 and 12 months as compared to 0 months (Figure 1B). However, when we compared 3 months against 6 and 12 months, the levels showed a decrease that remained constant throughout the 6 and 12 months comparison (Figure 1B).

### 2.2. Effects of Intense Exercise on White Blood Cells

**Figure 2:**
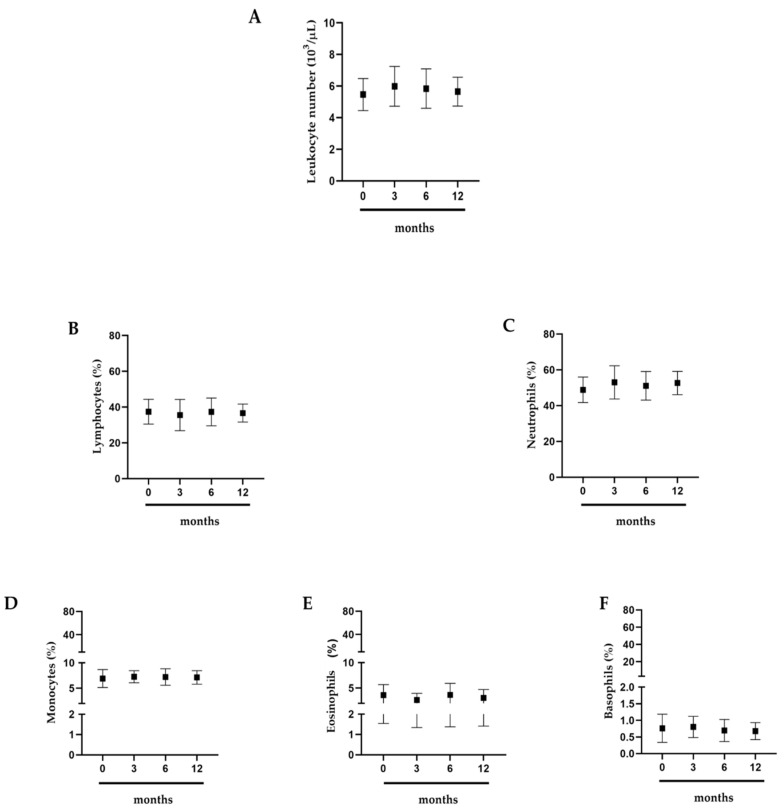
White blood cells. (**A**) Leukocytes; (**B**) lymphocytes; (**C**) neutrophils; (**D**) monocytes; (**E**) eosinophils; (**F**) basophils. Assessment of 15 professional basketball athletes at 0, 3, 6, and 12 months. The data are expressed as the means ± SDs. Significance was determined by the Student’s *t*-test.

To highlight how prolonged exercise affects athletes’ health, we evaluated the white blood cells of professional basketball athletes. It was interesting to note that leukocytes, lymphocytes, neutrophils, eosinophils, and basophils did not show significant variations (Figure 2A–F).

### 2.3. Measurements of Cortisol and Testosterone

**Figure 3:**
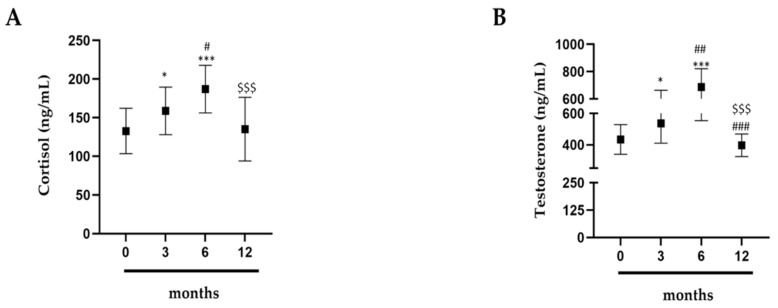
Hormonal dosages. (**A**) Cortisol; (**B**) testosterone. Evaluation of hormones in 15 professional basketball athletes at 0, 3, 6, and 12 months. The data are expressed as the means ± SDs. The significance was determined by the Student’s *t*-test: * (*p* < 0.05), (*p* < 0.01), and *** (*p* < 0.001) represent significance compared to 0 months; ^#^ (*p* < 0.05), ^##^ (*p* < 0.01), and ^###^ (*p* < 0.001) represent significance compared to 3 months; (*p* < 0.05), (*p* < 0.01), and ^$$$^ (*p* < 0.001) represent significance compared to 6 months.

To understand how physical activity influences anabolic and catabolic hormone trends, cortisol and testosterone titers were measured. In this analysis, both hormones were observed to increase when compared to 0 months (Figure 3A,B). Essentially, both hormones increased significantly between 0 and 6 months (Figure 3A,B); however, at 12 months, the values returned to baseline levels (Figure 3A,B).

### 2.4. Comparison of Inflammatory Indices

**Figure 4:**
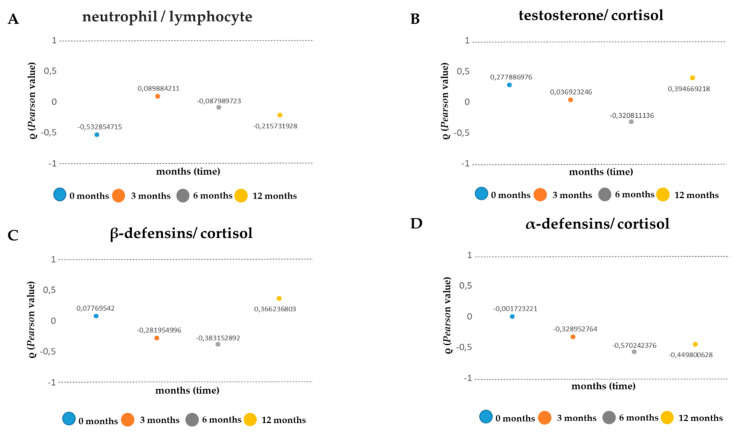
Inflammatory indices. (**A**) testosterone/cortisol ratios; (**B**) neutrophil/lymphocyte ratios; (**C**) beta-defensin/cortisol ratios; (**D**) alpha-defensin/cortisol ratios. Values obtained from the Pearson’s analysis are reported on the y-axis; time is reported on the x-axis.

To shed light on how professional physical activity influences cell/muscle “stress”, we evaluated four inflammatory indices [29,49] by analyzing and comparing neutrophil/lymphocyte ratios, testosterone/cortisol ratios, beta-defensin/cortisol ratios, and alpha-defensin/cortisol ratios. To emphasize the trends of these ratios over time, we used Pearson’s linear correlation coefficient (Table 1). In the analysis conducted, the neutrophil/lymphocyte ratio presented a moderate negative linear correlation in months 0 and 12, while at 3 and 6 months no correlation was observed (Table 1, Figure 4A). Other fascinating data were observed from the testosterone/cortisol ratios, where the two hormones demonstrated a negative linear correlation (Table 1, Figure 4B) at 6 months and a positive correlation at 12 months (Table 1, Figure 4B). For the beta-defensin/cortisol ratios, a negative linearity index was noted (Table 1, Figure 4C) between 3 and 6 months; in addition, at 12 months we noted a moderate positive correlation (Table 1, Figure 4C). Finally, the alpha-defensin/cortisol ratio exhibited a negative correlation between 0 and 12 months (Table 1, Figure 4D).

## 3. Discussion

Laboratory diagnostics in sports medicine are becoming increasingly important for the assessment and monitoring of athletes’ health [52,53]. Intense and continuous physical exercise, training, and competitions are known to induce metabolic adaptations, particularly in the serum concentrations of numerous biochemical parameters. These modifications reflect the changes that occur in the body in response to the intensity and duration of training exercises, as well as the specific stress of certain muscle groups and motor units during the execution of these exercises. These adaptations translate into alterations of specific parameters in terms of concentration and activity. Therefore, their identification could represent a new method of clinical evaluation.

We aimed to shed light on the biochemical, serological, and hematological parameter changes following exercise. We assessed several parameters, since the response to exercise-induced stress implies a complex involvement of organs and tissues and no studies currently exist in the literature regarding the integration of these different aspects. To make our observations, we collected peripheral blood samples from a group of professional athletes who had given informed consent. 

Following our observation of the results obtained, we highlighted that the levels of alpha- and beta-defensins (HNP1 and HBD-1, respectively) increased progressively with training intensification. These antimicrobial peptides have a fundamental role in the human innate immune system; indeed, they are involved in different inflammatory processes that respond to different human pathogens [54,55,56,57,58,59]. Their increase could coincide with athletes’ self-defense adaptations that protect against possible respiratory diseases that could affect them during competition seasons [20,21,22,23,24,25].

This study has shown that in the athletes examined, exercise does not cause alteration in the number of white blood cells. Increases in lymphocytes, neutrophils, monocytes, and basophils that are constant over time are known to act as sentinels for the improper functioning of the immune system or for the presence of an infectious disease that is prolonged and not properly eradicated [3,19,20,21,22,23,24,25].

The adaptations produced by the action of some hormones, such as cortisol and testosterone, have been particularly impressive. These two hormones titers allow the assessment of the athlete’s correct muscle condition. Our data showed that both hormones increase between months 0 and 6. However, the Pearson correlation shed light on the fact that their ratios have a negative linear correlation at 6 months. That is, at 6 months, the increase in cortisol is not directly proportional to the increase in testosterone. Since their increase is not directly proportional, it is possible to speak of a negative linear correlation, which is to say that an increase in testosterone does not correlate to a linear increase in cortisol. These data show a perfect balance between anabolic and catabolic metabolism [41,42,43,44,45,46,47,48,49]. In fact, it is known that increased testosterone levels may induce a reduction in the transforming growth factor beta (TGF-β) signal, which in turn reduces osteoprogenitor cells and increases lean muscle mass [59,60,61,62], thereby increasing anabolic effects. In fact, testosterone is an anabolic hormone (i.e., it binds proteins), thanks to which muscle growth occurs. At the same time, it provides energy to the muscles and accelerates the reconstruction of glycogen after the effort. The synergy of these mechanisms increases the athlete’s lean muscle mass and reduces the fat mass of the muscle.

Our study indicated that during intense and prolonged physical activity, the human body puts in place a series of defense and adaptation mechanisms to combat a prolonged stress state that could be harmful to both the respiratory and muscular systems. It is well known that competitive athletes follow a diet suited to sustaining their intense training schedules [63]. Often, this diet is enriched with food supplements and multivitamins in accordance with the antidoping rules in place. In this scenario, it would be useful to use supplements containing cycled and engineered analogues of alpha- and beta-defensins [63,64,65,66,67,68,69,70,71]. The use of these analogues could support the decreased inflammatory state to which the athlete is subjected, protecting them from any pathologies. In addition, as frequently reported in the literature, nutrition and, therefore, the intestinal microbiota are essential for the correct functionality of the whole body [57,72].

## 4. Materials and Methods

### 4.1. Experimental Approach

We evaluated leukocytes, antimicrobial peptides (i.e., alpha- and beta-defensins), cortisol (a catabolic hormone), and testosterone (an anabolic hormone) to evaluate the stresses undergone by professional basketball players and caused by physical exercise during competition season. The study was designed following the recommendations for clinical research contained in the Helsinki Declaration of the World Medical Association, and the protocol was approved by the Ethics Committee of the School of Medicine, University of Naples Federico II, protocol number 200/17.

### 4.2. Participants

This study examined professional basketball players (n = 15). The participants were all male and were informed about the research and the protocols used. The players had the following physical characteristics, reported as means ± SDs: age of 25 ± 6 years, weight of 92 ± 10 kg, and height of 195 ± 9 cm. None of the subjects smoked, drank alcohol, or consumed drugs known to alter the chemical parameters of the leukocyte or hormonal formula. All subjects followed a similar diet throughout the season and, above all, the same diet during the study. This was monitored continuously by the team doctors. The players followed the same training program, i.e., they trained every day for two sessions, with a morning session consisting of a gym workout for 2 h and an afternoon session consisting of basketball practice for 3 h. This training program was followed daily, except on the days of official games played during the season (two games per week).

### 4.3. Blood Sampling

Blood samples from competitive athletes were taken at 0 months in the preseason phase, 3 months after the start of the championship, 6 months after the start of the championship, and 12 months after the start of the championship. Leukocyte or hormone concentrations were evaluated using blood samples from the athletes. The parameters analyzed were leukocytes (4.8 × 10^3^ to 10.8 × 10^3^/µL), neutrophils (40–70%), lymphocytes (20–45%), monocytes (3–10%), eosinophils (0–6%), basophils (0–1.5%), cortisol (50–200 ng/mL), and testosterone (300–900 ng/dL). Blood and serum samples were taken in the morning, before training, for all athletes.

### 4.4. Biochemical Determinations

White blood cell counts were performed using the Siemens Advia 2120i hematology analyzer according to the manufacturer’s recommendations. Sera were analyzed for cortisol and testosterone concentrations by means of immunoassay procedures using the Immulite 2000 analyzer (Cortisol and Testosterone Immunoassay Kit; Siemens Healthineers, Malvern, PA, USA) according to the manufacturer’s recommendations.

### 4.5. Determination of α- and β-Defensins

Alpha- and beta-defensins were assessed in the participants’ sera using ELISA (Human DEFα1 and Human DEFB1 ELISA Kit; Elabscience, Buckingham, UK) according to the manufacturer’s recommendations. The sera were stored at −80 °C until use. The parameters analyzed were beta defensins (0.03125–2 µg/mL) and alpha defensins (0.03125–2 µg/mL).

### 4.6. Data Analysis and Statistics

All statistical analyses were performed using GraphPad Prism 8.4.0 (GraphPad Software Inc., La Jolla, CA, USA). Data were expressed as the means  ±  standard deviations. The Student’s *t*-test was used to compare the groups, with values of *p* < 0.05 considered significant. To evaluate the relationships between testosterone and cortisol, alpha-defensin and cortisol, beta-defensin and cortisol, and neutrophils and lymphocytes (to determine whether they could be used as indices of inflammation), Pearson’s linear correlation coefficient was used. Here, a value of +1 corresponded to a perfect positive linear correlation, 0 corresponded to an absence of a linear correlation, and −1 corresponded to a perfect negative linear correlation. In fact, the correlation index is +1 in the presence of a perfect positive linear correlation (i.e., Y = a + bX, b > 0), while it is -1 in the presence of a perfect negative linear correlation (i.e., Y = a + bX, b < 0).

## 5. Conclusions

The biochemical parameters analyzed in this study were used as indices to evaluate and monitor the physical conditions of athletes at different stages of a sports season in order to protect athletes’ health and also to prevent either the deterioration of athletes’ form or the occurrence of respiratory disease. This study lays the foundations for outlining a panel of specific markers in as much detail as possible, which then can be applied to individual athletes. Obtained results could be reported in a personalized file for each individual athlete, allowing athletic trainers and sports doctors to plan personalized training and recovery programs and, fundamentally, to monitor the health status of athletes in order to monitor the numerous effects induced by intense physical activity.

## Figures and Tables

**Table 1:** Pearson’s correlation (*ρ*) between neutrophils (N), lymphocytes (L), testosterone (T), cortisol (C), beta-defensins (B), and alpha-defensins (A) at different analyzed times. The Pearson index was considered significant for values between +1 and −1.

Variables	*P*			
	0 Months	3 Months	6 Months	12 Months
N vs. L	−0.53	0.08	−0.08	−0.21
T vs. C	0.28	0.04	−0.32	0.39
B vs. C	0.07	−0.28	−0.38	0.36
A vs. C	−0.001	−0.32	−0.57	−0.44
